# Trop-2-targeting tetrakis-ranpirnase has potent antitumor activity against triple-negative breast cancer

**DOI:** 10.1186/1476-4598-13-53

**Published:** 2014-03-10

**Authors:** Donglin Liu, Thomas M Cardillo, Yang Wang, Edmund A Rossi, David M Goldenberg, Chien-Hsing Chang

**Affiliations:** 1 IBC Pharmaceuticals, Inc., Morris Plains 07950, NJ, USA; 2 Immunomedics, Inc., Morris Plains 07950, NJ, USA; 3 Garden State Cancer Center, Center for Molecular Medicine and Immunology, Morris Plains 07950, NJ, USA

**Keywords:** Ranpirnase, Trop-2, DOCK-AND-LOCK™, ImmunoRNase, Breast cancer

## Abstract

**Background:**

Ranpirnase (Rap) is an amphibian ribonuclease with reported antitumor activity, minimal toxicity, and negligible immunogenicity in clinical studies, but the unfavorable pharmacokinetics and suboptimal efficacy hampered its further clinical development. To improve the potential of Rap-based therapeutics, we have used the DOCK-AND-LOCK™ (DNL™) method to construct a class of novel IgG-Rap immunoRNases. In the present study, a pair of these constructs, (Rap)_2_-E1-(Rap)_2_ and (Rap)_2_-E1*-(Rap)_2_, comprising four copies of Rap linked to the C_H_3 and C_K_ termini of hRS7 (humanized anti-Trop-2), respectively, were evaluated as potential therapeutics for triple-negative breast cancer (TNBC).

**Methods:**

The DNL-based immunoRNases, (Rap)_2_-E1-(Rap)_2_ and (Rap)_2_-E1*-(Rap)_2_, were characterized and tested for biological activities in vitro on a panel of breast cancer cell lines and in vivo in a MDA-MB-468 xenograft model.

**Results:**

(Rap)_2_-E1-(Rap)_2_ was highly purified (>95%), exhibited specific cell binding and rapid internalization in MDA-MB-468, a Trop-2-expressing TNBC line, and displayed potent in vitro cytotoxicity (EC_50_ ≤ 1 nM) against diverse breast cancer cell lines with moderate to high expression of Trop-2, including MDA-MB-468, BT-20, HCC1806, SKBR-3, and MCF-7. In comparison, structural counterparts of (Rap)_2_-E1-(Rap)_2_, generated by substituting hRS7 with selective non-Trop-2-binding antibodies, such as epratuzumab (anti-CD22), were at least 50-fold less potent than (Rap)_2_-E1-(Rap)_2_ in MDA-MB-468 and BT-20 cells, both lacking the expression of the cognate antigen. Moreover, (Rap)_2_-E1-(Rap)_2_ was less effective (EC_50_ > 50 nM) in MDA-MB-231 (low Trop-2) or HCC1395 (no Trop-2), and did not show any toxicity to human peripheral blood mononuclear cells. In a mouse TNBC model, a significant survival benefit was achieved with (Rap)_2_-E1*-(Rap)_2_ when given the maximal tolerated dose.

**Conclusions:**

A new class of immunoRNases was generated with enhanced potency for targeted therapy of cancer. The promising results from (Rap)_2_-E1-(Rap)_2_ and (Rap)_2_-E1*-(Rap)_2_ support their further investigation as a potential treatment option for TNBC and other Trop-2-expressing cancers.

## Background

Breast cancer is the leading cause of cancer deaths in women and the second most common cancer worldwide after lung cancer [1]. In the USA, about 232,340 new cases of this cancer will be diagnosed and about 39,620 women will die from this disease in 2013 [2]. During the past two decades, important advances have been made in the treatment of hormone receptor (HR)-positive [3,4] and human epidermal growth factor receptor type 2 (HER2)-overexpressing breast cancers [5,6]. However, effective therapies for patients with triple-negative breast cancer (TNBC), which lacks the expression of estrogen receptor (ER), progesterone receptor (PR) and HER2, are still urgently needed [7]. TNBC, afflicting approximately 12 to 17% of women with breast cancer [8], is a heterogeneous disease with varying prognoses according to molecular, pathological, and clinical factors [9,10]. Because of the absence of clear targets like HER2, ER, and PR, chemotherapy with doxorubicin plus cyclophosphamide followed by paclitaxel is recommended as the standard of care for systemic treatment of TNBC in the adjuvant setting [11], whereas for patients with metastatic disease, there is no standard first-line agent. To date, various approved agents, including platinum-based compounds, ixabepilone, erlotinib, bevacizumab, cetuximab, and several investigative drugs, in particular, inhibitors of PARP, tyrosine kinases, or mTOR, are being evaluated alone or in combination in different phases of clinical development for TNBC [11]. An arduous challenge in TNBC chemotherapy is primary or acquired drug resistance, resulting in incomplete response, relapse and poor survival.

Rap is a single-chain protein of 104 amino acids (MW ~12 kDa) originally isolated from the oocytes of *Rana pipiens*, a Northern leopard frog [12]. Rap exhibits cytostatic and cytotoxic effects on a variety of tumor cell lines in vitro [13], as well as antitumor activity in vivo [14]. The amphibian ribonuclease enters cells via AP-2/clathrin-mediated endocytosis [15] and once internalized into the cytosol, it selectively degrades tRNA [16], thereby inhibiting protein synthesis and inducing apoptosis [17]. In addition, Rap was shown to enhance both the in vitro and in vivo antitumor activity of vincristine against HT-29 human colorectal cancer cells that had been rendered multidrug-resistant by overexpressing the *mdr1* gene [18], and induce caspase-independent cell death in both drug-sensitive and -resistant neuroblastoma cells and tumor xenografts [19]. Clinical studies of Rap in patients with unresectable malignant mesothelioma showed a significant impact on the survival of patients treated with doxorubicin plus Rap compared to doxorubicin alone [20], and a dose-limiting renal toxicity that was reversible upon discontinuation of treatment [21]. Notably, an earlier Phase I trial of Rap in patients with solid cancers reported a lack of untoward immune response upon repeated weekly injections [22].

Trop-2, also known as EGP-1 (epithelial glycoprotein-1), is a cell-surface glycoprotein overexpressed by a variety of epithelial carcinomas relative to corresponding normal tissues [23]. The expression of Trop-2 was shown to be necessary for tumorigenesis and invasiveness of colon cancer cells, which could be reduced effectively with a polyclonal antibody against the extracellular domain of Trop-2 [24]. More recently, the biological function of Trop-2 in promoting self-renewal and hyperplasia in the prostate was attributed to the accumulation in the nucleus of the intracellular domain of Trop-2 following its cleavage via regulated intramembrane proteolysis [25]. Because of the well-documented clinical evidence in breast cancer [26], colorectal cancer [27,28] and other cancers [29] that overexpressed Trop-2 is associated with increased tumor aggressiveness, metastasis, and decreased patient survival, there is a growing interest in Trop-2 as a therapeutic target for solid cancers [30]. For example, the humanized anti-Trop-2 monoclonal antibody (mAb), hRS7, is currently under clinical investigation as a drug delivery moiety for patients with advanced epithelial cancers (NCT01631552).

The DOCK-AND-LOCK™ (DNL™) method [31-33] is a platform technology for production of multivalent, multifunctional conjugates by utilizing the naturally occurring interaction between the dimerization and docking domain (DDD) of cAMP-dependent protein kinase A (PKA) and the anchoring domain (AD) of an A-kinase anchoring protein (AKAP). The established strategy involves the use of a specific pair of DDD and AD peptides, termed DDD2 and AD2, to generate two distinct modules, which upon mixing under redox conditions, self-assemble into a DNL conjugate with retained activity and defined composition. We previously reported the antitumor activity of a novel immunotoxin, designated Rap(Q)-hRS7, in Trop-2-expressing cancer cells [34]. The recombinantly-produced Rap(Q)-hRS7 comprises two molecules of Rap(p.N69Q) or N69Q-Rap, a variant of Rap with the putative N-glycosylation site removed by replacing the 69^th^ residue of asparagine (N69) with glutamine (Q), each fused to a light chain of hRS7 at the amino terminus [34].

**Figure 1:**
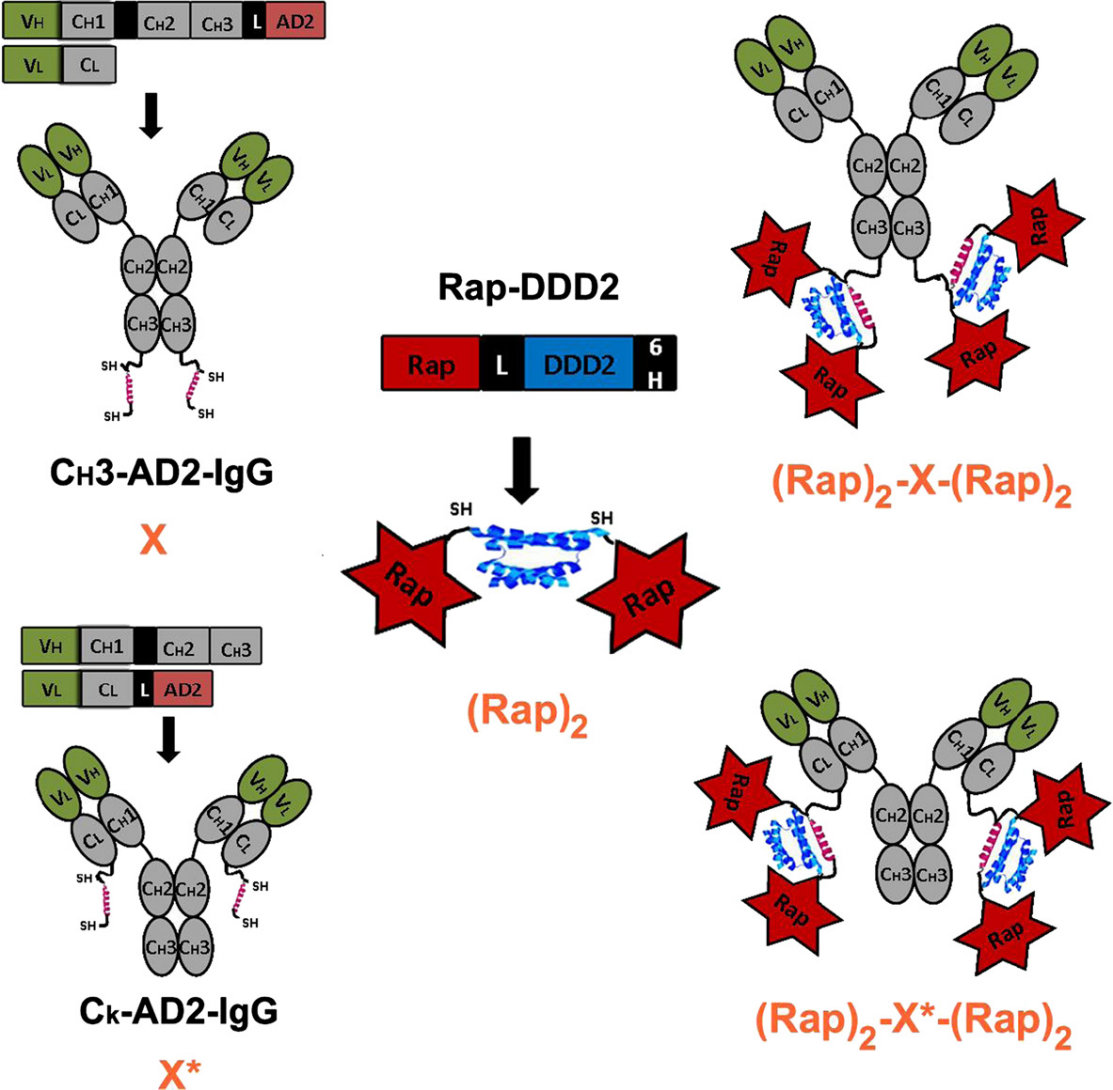
**Schematic of expression cassettes and cartoon structures of C**_**H**_**3-AD2-IgG (or X), C**_**k**_3-AD2-IgG (or X*), Rap-DDD2, (Rap)_**2**_**, (Rap)**_**2**_**-X-(Rap)**_**2**_**, and (Rap)**_**2**_-X*-(Rap)_**2**_**.** DDD2 and AD2 are shown in blue and red, respectively. SH, V, C, G, L, and 6H represent sulfhydryl groups, variable domain (green), constant region (gray), hinge, linker (GSGGGGSGG), and His tag (HHHHHH), respectively.

To further explore the potential of Rap-based immunotoxins, we applied the DNL method to site-specifically tether a dimerized Rap, denoted as (Rap)_2_, where (Rap) represents Rap-DDD2, at each of the carboxyl termini of either the heavy chain (the C_H_3–format) or the light chain (the C_K_-format) of a humanized IgG, resulting in a class of novel immunoconjugates (Table 1) with tetrakis Rap (Figure 1). As exemplified herein by (Rap)_2_-E1-(Rap)_2_, comprising four copies of Rap linked to the AD2-fused C_H_3 termini of hRS7 IgG (denoted as E1), and by (Rap)_2_-E1*-(Rap)_2_, comprising four copies of Rap linked to the AD2-fused C_K_ termini of hRS7 IgG (denoted as E1*), these DNL-based immunoRNases could offer a distinct advantage over their recombinantly-produced fusion counterparts, such as Rap(Q)-hRS7, in improved potency against targeted cancer cells in vitro and tumor xenografts in vivo.

**Table 1:** DNL-Rap conjugates used in the study

**Code**	**Target**	**AD2-module**	**DDD2-module**
(Rap)_2_-E1-(Rap)_2_	Trop-2	C_H_3-AD2-IgG-hRS7	Rap-DDD2
(Rap)_2_-22-(Rap)_2_	CD22	C_H_3-AD2-IgG-hLL2	Rap-DDD2
(Rap)_2_-20-(Rap)_2_	CD20	C_H_3-AD2-IgG-hA20	Rap-DDD2
(Rap)_2_-74-(Rap)_2_	CD74	C_H_3-AD2-IgG-hLL1	Rap-DDD2
(Rap)_2_-C2–(Rap)_2_	HLA-DR	C_H_3-AD2-IgG-hL243	Rap-DDD2
(Rap)_2_-14-(Rap)_2_	CEACAM5	C_H_3-AD2-IgG-hMN-14	Rap-DDD2
(Rap)_2_-E1*-(Rap)_2_	Trop-2	C_K_-_-_AD2-IgG-hRS7	Rap-DDD2
(Rap)_2_-22*-(Rap)_2_	CD22	C_K_-AD2-IgG-hLL2	Rap-DDD2

## Results

### Preparation and characterization of DNL-Rap conjugates

Each DNL-Rap conjugate (Table 1) was obtained with a general protocol involving (i) expression of the respective IgG-AD2 module in myeloma cells, with purification by protein A-based MabSelect affinity chromatography; (ii) expression of Rap-DDD2 in *E. coli* as inclusion bodies, purified by immobilized metal affinity chromatography under denaturing conditions, and subsequently refolded; (iii) combination of IgG-AD2 with Rap-DDD2 under redox conditions; and (iv) purification of the resulting DNL-Rap conjugate by MabSelect chromatography. Representative results on the SDS-PAGE, size exclusion high-performance liquid chromatography (SE-HPLC) and dynamic light scattering analyses demonstrating the molecular purity and integrity of DNL-Rap conjugates are shown for (Rap)_2_-E1-(Rap)_2_, (Rap)_2_-E1*-(Rap)_2_, (Rap)_2_-22-(Rap)_2_, and (Rap)_2_-22*-(Rap)_2_ (Additional file 1: Figure S1A-E), and summarized below. On non-reducing gel, (Rap)_2_-E1-(Rap)_2_ was detected as a predominant band (Additional file 1: Figure S1A; lane 6) having the expected size between the monomer and dimer of C_H_3-AD2-IgG-hRS7 (Additional file 1: Figure S1A; lane 5); under reducing conditions, only three bands (Additional file 1: Figure S1A; lane 3) were evident, which correspond to the three constituents of (Rap)_2_-E1-(Rap)_2_, namely, Rap-DDD2 (Additional file 1: Figure S1A; lane 1), and the heavy (H) and light (L) chains from C_H_3-AD2-IgG- hRS7 (Additional file 1: Figure S1A; lane 2), indicating the absence of non-product proteins. SE-HPLC analyses of (Rap)_2_-E1-(Rap)_2_, C_H_3-AD2-IgG-hRS7, and Rap-DDD2 revealed that each existed as a major peak with retention times consistent with their calculated masses (Additional file 1: Figure S1B). Dynamic light scattering analysis of (Rap)_2_-E1-(Rap)_2_ showed an average diameter of 15.83 nm, with a width of 6.36 nm (Additional file 1: Figure S1C). Whereas the C_K_- and C_H_3-formats of DNL conjugates displayed similar SE-HPLC profiles (Additional file 1: Figure S1D, (Rap)_2_-E1*-(Rap)_2_ vs. (Rap)_2_-E1-(Rap)_2_), they were distinguishable on reducing SDS-PAGE, with the former having a faster moving H chain and a slower moving L chain (Additional file 1: Figure S1E, lane 2 vs. lane 4). The molecular purity of (Rap)_2_-22*-(Rap)_2_, as well as that of (Rap)_2_-22-(Rap)_2_, was demonstrated also by reducing SDS-PAGE in Additional file 1: Figure S1E in lanes 2 and 4, respectively, showing the presence of only three bands in either lane. Further, the three bands in lane 2 matched the single band of Rap-DDD2 (lane 3) and the double bands of the H and L chains from C_K_-AD2-IgG-hLL2 (lane 1). Likewise, the three bands in lane 4 matched the single band of Rap-DDD2 (lane 3) and the two double bands of H and L chains from C_H_3-AD2-IgG-hLL2 (lane 5).

### Trop-2 expression on breast cancer cells

The cell surface expression of Trop-2 on a variety of human breast cancer cell lines was evaluated by flow cytometry using hRS7 IgG as the primary antibody. Seven cell lines were examined, including five basal-like, triple-negative subtypes (MDA-MB-468, MDA-MB-231, BT-20, HCC1806, and HCC1395), one luminal B, HER2-negative subtype (MCF-7), and one HER2-positive subtype (SKBR-3). HCC1395 was the only cell line found to be negative, and MDA-MB-231 showed minimal expression (Median fluorescence intensity, MFI = 10.37), while all other lines had moderate (MFI = 40-100) to high (MFI = 100-400) levels of Trop-2 (Additional file 2: Figure S2 and Additional file 3: Table S1).

### In vitro cytotoxicity

**Figure 2:**
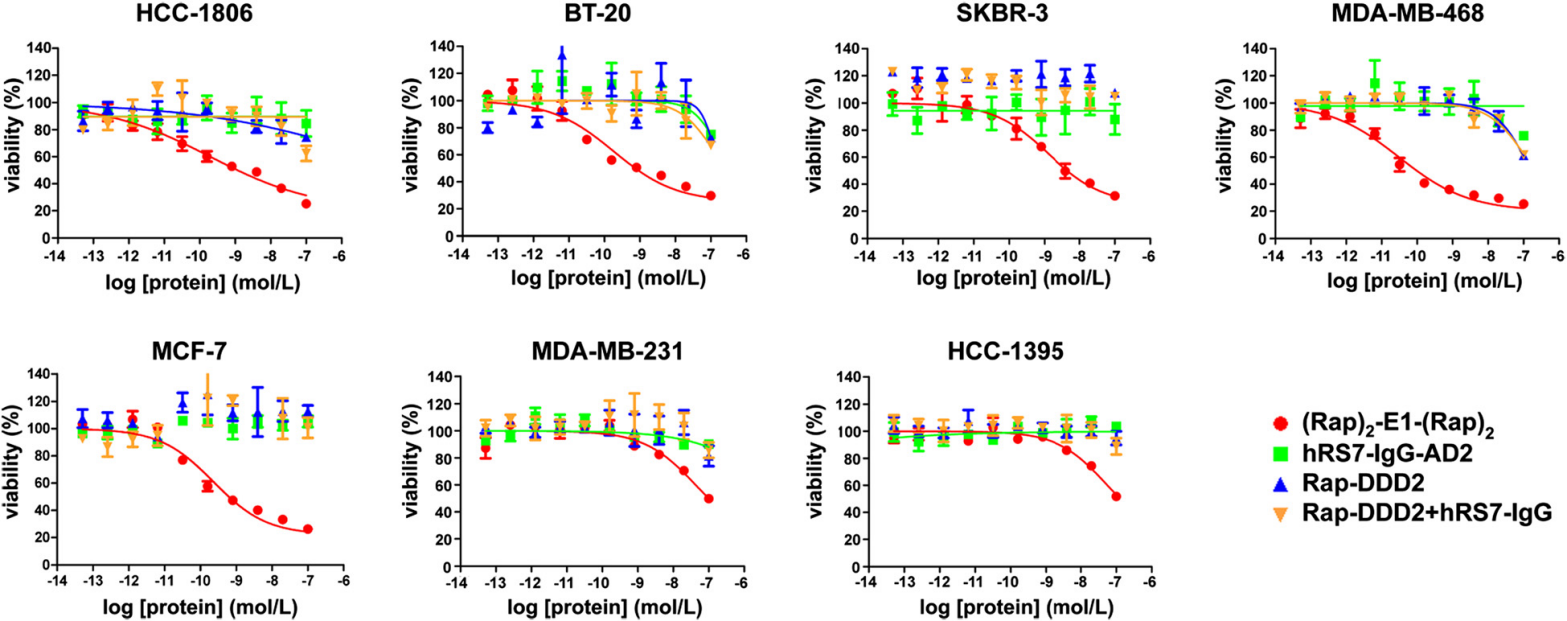
**In vitro cytotoxity of (Rap)**_**2**_**-E1-(Rap)**_**2 **_**in selected breast cancer cell lines.** Cells were cultured for 4 days in the presence of (Rap)_2_-E1-(Rap)_2_, C_H_3-AD2-IgG-hRS7, Rap-DDD2, or a combination of hRS7 IgG and Rap-DDD2 at indicated concentrations. Viable cells were determined with the MTS assay, and the results relative to untreated cells were plotted against log of concentrations. Half maximal effective concentration (EC_50_) values were determined using non-linear regression sigmoidal dose–response curve of Prism software.

**Figure 3:**
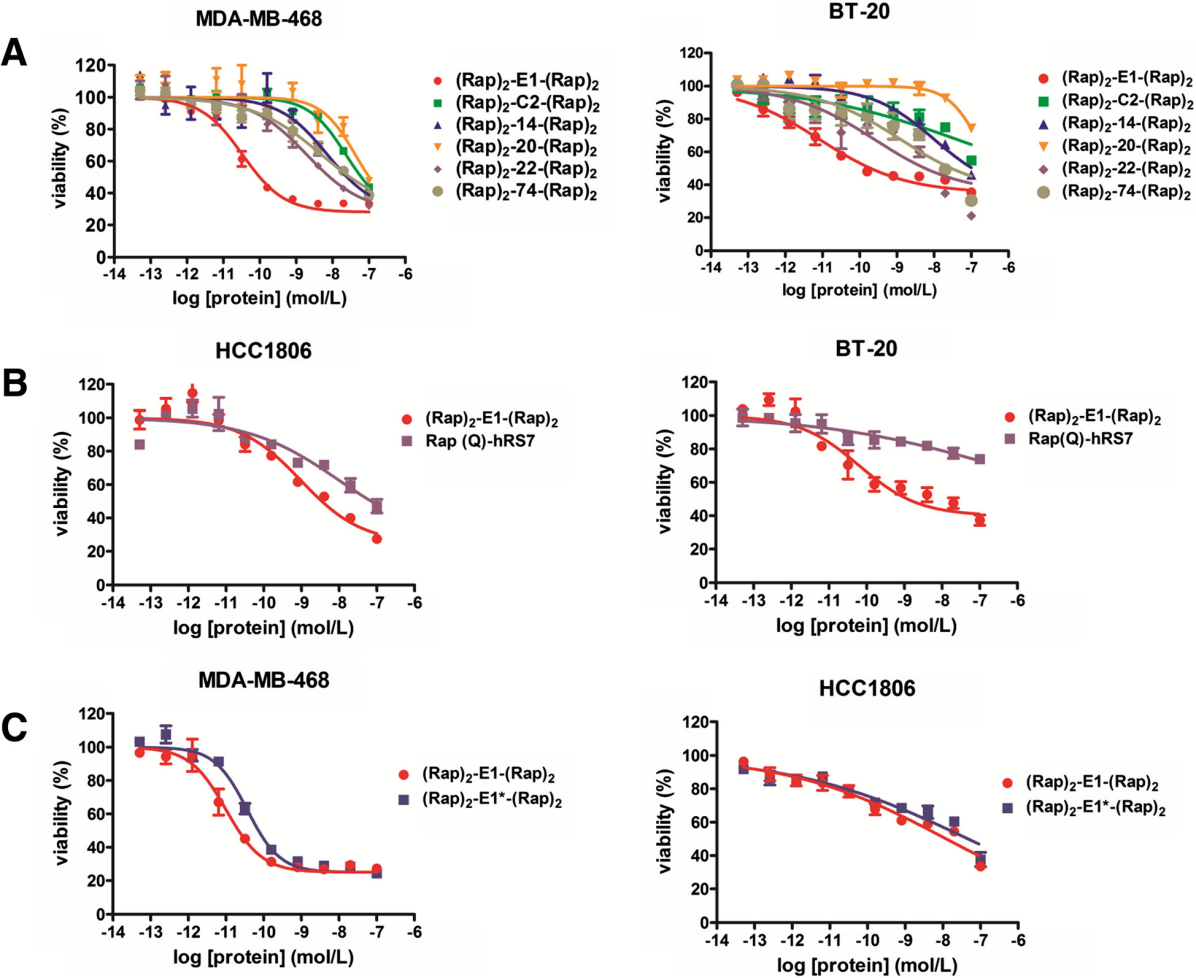
**Selection of (Rap)**_**2**_**-E1-(Rap)**_**2 **_**for TNBC.** The potency of (Rap)_2_-E1-(Rap)_2_ was compared: **A**, with five non-Trop-2-targeting DNL-Rap conjugates in MDA-MB-468 and BT-20; **B**, with Rap(Q)-hRS7 in BT-20 and HCC1806; and **C**, with (Rap)_2_-E1*-(Rap)_2_ in HCC1806 and MDA-MB-468. (Rap)_2_-C2–(Rap)_2_, (Rap)_2_-14-(Rap)_2_, (Rap)_2_-20-(Rap)_2_, (Rap)_2_-22-(Rap)_2_, and (Rap)_2_-74-(Rap)_2_ are five counterparts of (Rap)_2_-E1-(Rap)_2_, generated by substituting hRS7 with humanized antibodies that targets HLA-DR, CEACAM5, CD20, CD22, and CD74, respectively.

Based on the results of a 4-day MTS assay (Figure 2), (Rap)_2_-E1-(Rap)_2_ exhibited subnanomolar to nanomolar EC_50_ values for MDA-MB-468 (0.03 nM), MCF-7 (0.1 nM), BT-20 (0.18 nM), HCC1806 (0.19 nM), and SKBR-3 (1.29 nM), all of which express moderate to high levels of Trop-2. In comparison, (Rap)_2_-E1-(Rap)_2_ inhibited proliferation of MDA-MB-231 (low Trop-2) or HCC1395 (negative Trop-2) only at higher concentrations (EC_50_ > 50 nM). In all cell lines tested, either module (C_H_3-AD2-IgG-hRS7, Rap-DDD2) alone or the combination of the parental hRS7 IgG with Rap-DDD2 showed negligible cytotoxicity. Besides breast cancer cell lines, potent cytotoxicity with subnanomolar EC_50_ values was observed for (Rap)_2_-E1-(Rap)_2_ in Trop-2-expressing cell lines of other cancers (Additional file 4: Figure S3), including prostate cancer (MDA PCa 2b, PC-3), lung cancer (Calu-3), pancreatic cancer (BxPC-3), and cervical cancer (ME-180), but not in Trop-2-negative cell lines, such as 22Rv1 (prostate cancer). Further studies showed (Rap)_2_-E1-(Rap)_2_ to be the most effective in inhibiting the proliferation of MDA-MB-468 or BT-20 compared to other DNL-Rap conjugates that do not target Trop-2 (Figure 3A). The observed EC_50_ values from the current and previous studies also indicate (Rap)_2_-E1-(Rap)_2_ to be considerably more cytotoxic than Rap(Q)-hRS7 in HCC-1806 and BT-20 (Figure 3B), as well as in MDA-MB-468, MCF-7 and SKBR-3 (Table 2). Moreover, the potency of (Rap)_2_-E1*-(Rap)_2_, the C_K_-counterpart of (Rap)_2_-E1-(Rap)_2_, was comparable to (Rap)_2_-E1-(Rap)_2_ in HCC-1806, but appeared to be reduced in potency in MDA-MB-468 (Figure 3C).

**Table 2:** **Comparison of in vitro potency of (Rap)**_**2**_**-E1-(Rap)**_**2 **_**vs. Rap(Q)-hRS7**

**Agents**	**EC**_**50 **_**(nM)**^**a**^						
	**MDA-MB-468**	**MCF-7**	**SKBR-3**	**HCC1806**	**BT-20**	**MDA-MB-231**	**HCC1395**
(Rap)_2_-E1-(Rap)_2_	0.03	0.1	1.29	0.19	0.18	>50	~100
Rap(Q)-hRS7	3.8^b^	>100^b^	>100^b^	10.1	>100	ND^c^	ND^c^

^a^Half maximal effective concentration; ^b^From previous results [34]; ^c^Not determined.

### Cell binding and internalization of (Rap)_2_-E1-(Rap)_2_

**Figure 4:**
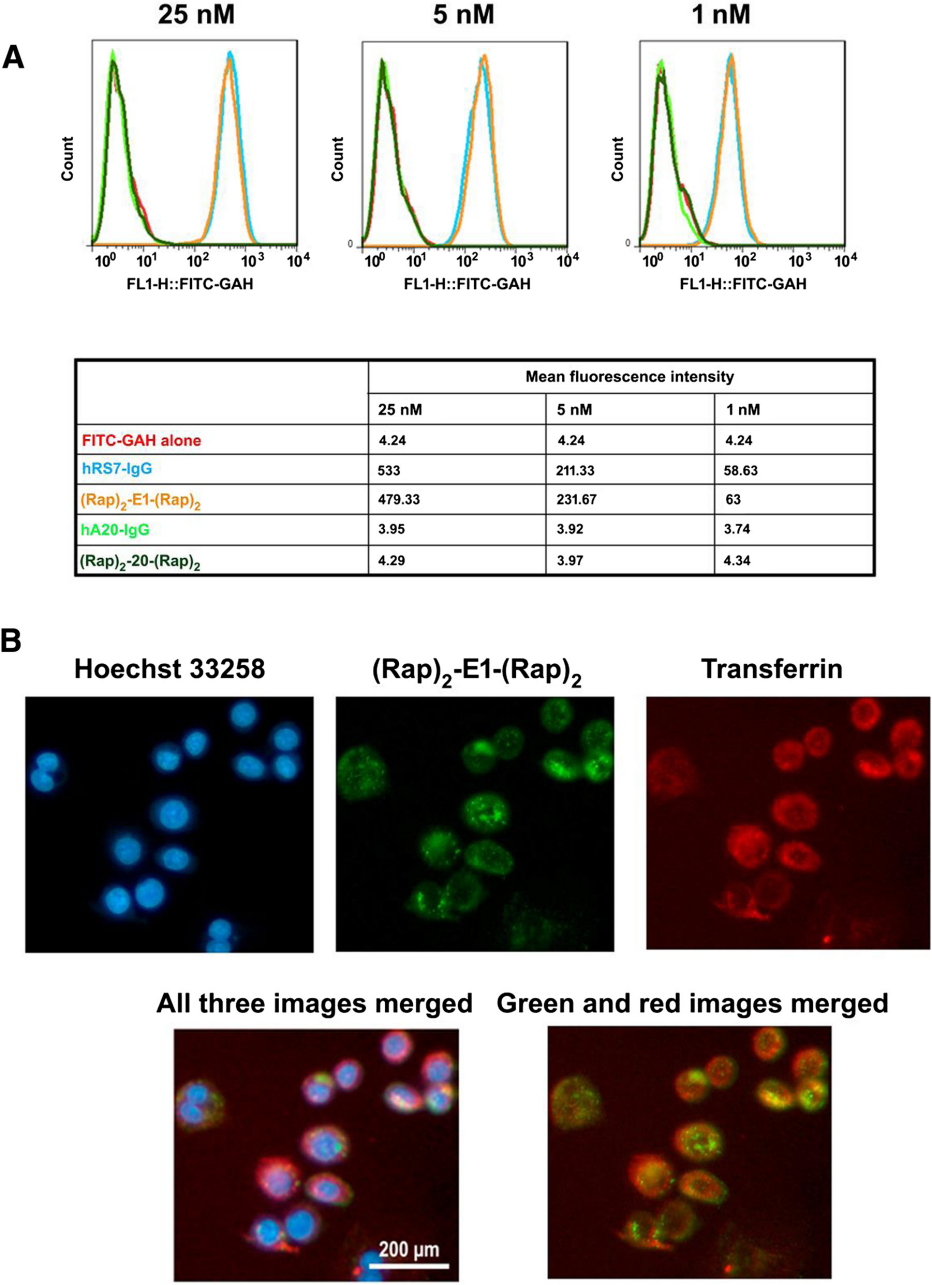
**Evaluation of binding and internalization of (Rap)**_**2**_**-E1-(Rap)**_**2 **_**in MDA-MB-468. A**, flow cytometric analyses showing equivalence in binding of (Rap)_2_-E1-(Rap)_2_ and hRS7 IgG to MDA-MB-468 at three concentrations (1, 5 and 25 nM). The CD20-targeting (Rap)_2_-20-(Rap)_2_ and hA20 served as negative controls; **B**, following incubation, pH 2.5 treatment, and fixation as described in the Methods, cells were stained with GAH- Alexa Fluor 488 (green) to detect intracellular (Rap)_2_-E1-(Rap)_2_, and also with Hoechst 33258 (blue) and human transferrin-conjugated Alexa Fluor 568 (red) to reveal the nucleus and endosomes, respectively.

We compared the binding affinity of (Rap)_2_-E1-(Rap)_2_ with that of parental hRS7-IgG for MDA-MB-468 cells. As shown in Figure 4A, (Rap)_2_-E1-(Rap)_2_ and hRS7-IgG bound equivalently to MDA-MB-468 at all three concentrations examined, indicating the avidity of (Rap)_2_-E1-(Rap)_2_ for Trop-2 is not compromised. We also examined the intracellular distribution of (Rap)_2_-E1-(Rap)_2_ in MDA-MB-468, and found it was effectively internalized at 37°C after 2-h incubation, and existed mostly as *punctas* within the cytoplasm (Figure 4B).

### Minimal toxicity of (Rap)_2_-E1-(Rap)_2_ on hematological cells

The toxicity of (Rap)_2_-E1-(Rap)_2_ to hematological cells was assessed on human peripheral blood mononuclear cells (PBMC) obtained from two healthy donors, following treatment with (Rap)_2_-E1-(Rap)_2_ at 0.1, 1, and 10 nM for 18 h. Treatment with (Rap)_2_-E1-(Rap)_2_ had nearly the same amount of apoptotic and dead cells as the untreated control (Additional file 5: Figure S4), indicating that (Rap)_2_-E1-(Rap)_2_ is not toxic to PBMC at concentrations up to 10 nM.

### MTD in nude mice

Among the four groups (20, 40, 60 or 80 μg) tested, only those mice that received the four injections of (Rap)_2_-22*-(Rap)_2_ at 20 μg survived without acute toxicity or death. One of the three animals lost ~12% of its body weight by day 4, but this was only transient, and by day 7 had regained much of this lost weight. All three mice survived to the end of the study on day 56. The maximum tolerated dose (MTD) of a DNL-Rap conjugate was therefore defined as 20 μg, administered i.v. every four days for four times (q4dx4). Although we have not formally determined the MTD of (Rap)_2_-E1*-(Rap)_2_ or other DNL-Rap conjugates in similar studies, subsequent experiments in tumor-bearing mice indicated that the animals also tolerated (Rap)_2_-E1*-(Rap)_2_ or (Rap)_2_-20-(Rap)_2_ when given the dosing regimen of 20 μg i.v., q4dx4.

### In vivo activity

We selected (Rap)_2_-E1*-(Rap)_2_ for in vivo studies because the C_K_-based conjugates exhibited superior Fc-effector functions in vitro, as well as improved pharmacokinetics, stability, and activity in vivo, as demonstrated for two types of DNL conjugates having a similar structure to IgG-Rap; namely, a bispecific hexavalent antibody comprising a pair of dimeric Fab linked to an IgG, and a multivalent immunocytokine comprising a pair of dimeric IFN-α linked to an IgG [35].

**Figure 5:**
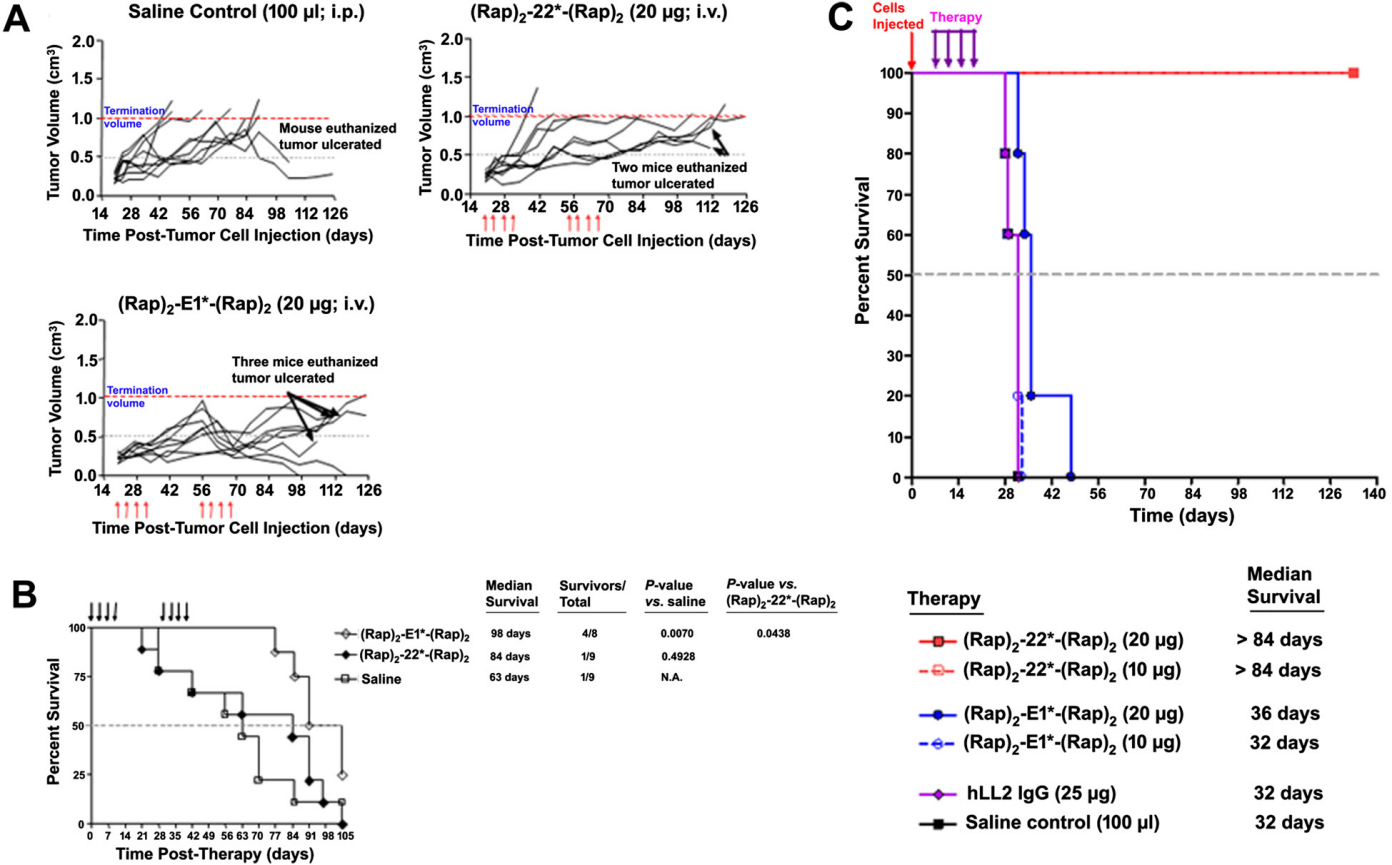
**In vivo studies of DNL-Rap conjugates.** (Rap)_2_-E1*-(Rap)_2_ was compared with (Rap)_2_-22*-(Rap)_2_ in a MDA-MB-468 xenograft model, showing tumor growth in individual mice in **A**, and survival curves in **B**; (Rap)_2_-22*-(Rap)_2_ was compared with (Rap)_2_-E1*-(Rap)_2_ in a disseminated Daudi model, showing survival curves in **C**.

In the TNBC model of mice bearing MDA-MB-468 tumors, all mice tolerated the two cycles of treatment with no mouse losing more than 9% of starting body weight during dosing (Additional file 6: Figure S5). Based on area under the curve (AUC) determined on day 48, (Rap)_2_-E1*-(Rap)_2_ significantly inhibited tumor growth compared to the saline control (Figure 5A; *P* = 0.0254). In addition, a significant survival benefit (Figure 5B), as indicated by the median survival time, was obtained with the (Rap)_2_-E1*-(Rap)_2_ group (98 days) when compared to either the saline group (63 days; *P* < 0.0070) or the (Rap)_2_-22*-(Rap)_2_ group (84 days; *P* < 0.0438). Notably, 2 of 8 mice in the (Rap)_2_-E1*-(Rap)_2_ group were tumor-free when the experiment ended on day 105.

To further demonstrate the targeting specificity of (Rap)_2_-E1*-(Rap)_2_ vs. (Rap)_2_-22*-(Rap)_2_, we performed another study to compare their potency in a CD22-expressing, but Trop-2-negative, tumor model. Mice bearing disseminated Burkitt-NHL (Daudi) were treated 7 days after the animals were administered the cells to ensure a large tumor-burden at the time of therapy initiation (Figure 5C). All mice in the saline control group succumbed to disease progression by day 32 (median = 32 days). Likewise, mice treated with the MTD of (Rap)_2_-E1*-(Rap)_2_ were all dead by day 48 (median = 36 days). Conversely, all mice that received the MTD of (Rap)_2_-22*-(Rap)_2_ or half this dose were still alive and tumor-free at the time the experiment was ended on day 84. Overall, treatment with (Rap)_2_-22*-(Rap)_2_ resulted in a significant survival benefit when compared to the saline or treatment control (*P* < 0.0034).

## Discussion

Rap is a promising antitumor toxin with several attractive properties, including minimal toxicity, negligible immunogenicity, and the potential to overcome multiple drug resistance via unusual mechanisms of internalization and cytotoxicity. Over the years, we have made considerable progress to enhance the potency of Rap by targeting it to cancer cells expressing CD22, CD74 and Trop-2, as exemplified by LL2-onconase [36], Rap-hLL1 [37], and Rap(Q)-hRS7 [34], respectively, but have also encountered difficulty in scaled-up production of these prototypes due to relatively low productivity of Rap-hLL1 or Rap(Q)-hRS7 in mammalian cell cultures. We believe the DNL™ platform technology, which conveniently tethers four copies of Rap to the C-terminus of different IgG modules, has key production advantages for Rap-based immunotoxins. Moreover, these DNL-Rap conjugates ensure targeted delivery of Rap with significantly improved potency in cancer cells expressing the cognate antigens, as exemplified by (Rap)_2_-E1-(Rap)_2_ in the current study with breast cancer cell lines. It is noteworthy that in MDA-MB-468 cells with a moderate level of Trop-2, the cytotoxicity of the DNL-generated (Rap)_2_-E1-(Rap)_2_ (EC_50_ = 0.03 nM) was 100-fold more potent than the recombinant Rap(Q)-hRS7 (EC_50_ = 3.8 nM), and 3,000-fold more potent than unconjugated Rap (EC_50_ > 100 nM). On the other hand, (Rap)_2_-E1-(Rap)_2_ inhibited the proliferation of Trop-2-negative HCC1395 cells only at much higher concentrations (EC_50_ > 50 nM) and yet was not toxic to PBMCs, indicating the likely existence of a relatively large therapeutic window.

We reported previously that in MDA-MB-468 cells, Rap(Q)-hRS7 co-localized with human transferrin in the endosomes after a 2-h incubation at 37°C [34], whereas in the current study under the same conditions, (Rap)_2_-E1-(Rap)_2_ did not co-localize with human transferrin in the endosomes, suggesting a different internalization pathway, which may explain its greater potency in MDA-MB-468 than observed for Rap(Q)-hRS7.

We noted that unlike the bispecific hexavalent antibodies, for which the C_K_-format was demonstrated to be superior to the C_H_3-format [35], the potential advantage of a C_K_-format of DNL-Rap vs. its C_H_3-counterpart is not readily apparent from the present data, and will require further studies.

The potency and specificity of DNL-Rap conjugates for targeted cancer therapy is also supported by the statistically significant antitumor activity of (Rap)_2_-E1*-(Rap)_2_ in suppressing growth of MDA-MB-468 xenografts, and by the observed cure of all five mice with disseminated Daudi lymphoma following treatment with four 20-μg doses of (Rap)_2_-22*-(Rap)_2_ given 4 days apart, both of which should reduce the potential concern that the larger size of DNL-Rap conjugates would negatively impact their in vivo efficacy, particularly in solid cancer, due to poor penetration.

Although hRS7 binds to selected human epithelial cells, recent studies with a drug conjugate of hRS7 in cynomolgus monkeys, whose tissues cross-react with hRS7, showed that tolerated doses occurred at clinically-relevant concentrations, and that dose-limiting toxicities to normal tissues were no different from those reported previously with the free drug [38]. These data suggest that having Rap attached to hRS7 may not add substantially its toxicity to normal tissues, and that (Rap)_2_-E1-(Rap)_2_ or (Rap)_2_-E1*-(Rap)_2_ may be tolerated at clinically-relevant concentrations. In addition, because the structural motif (x)D(y) identified to be responsible for the binding of ricin A-chain or interleukin-2 to endothelial cells is absent in the native sequence of Rap, and hRS7 is not cross-reactive with human endothelial cells, we consider the likelihood of (Rap)_2_-E1-(Rap)_2_ or (Rap)_2_-E1*-(Rap)_2_ causing vascular leak syndrome as remote. Regarding the immunogenicity of DNL-Rap conjugates, it needs to be addressed in a relevant species as well as in future human studies.

## Conclusions

We have generated a new class of immunoRNases and demonstrated their potential for targeted therapy of cancer. These promising results warrant further studies to advance the development of (Rap)_2_-E1-(Rap)_2_ or (Rap)_2_-E1*-(Rap)_2_, in particular, and other DNL-Rap conjugates, in general, as potential novel therapeutics for triple-negative breast cancer or other Trop-2-expressing cancers.

## Methods

### Antibodies and reagents

Humanized antibodies including hA20 (anti-CD20, veltuzumab), hLL2 (anti-CD22, epratuzumab), and hRS7 (anti-Trop-2) were obtained in-house. FITC-labeled goat anti-human IgG (FITC-GAH) was purchased from Jackson ImmunoResearch Laboratories (West Grove, PA). Annexin V Alexa Fluor 488 conjugate and 7-AAD were purchased from Life Technologies Corporation (Grand Island, NY).

### Expression and purification of DNL modules

To produce the Rap-DDD2 module used for DNL conjugation, a DNA fragment encoding Rap and a GGGGS linker sequence was amplified by PCR from *rPRL2#26* plasmid [34] and inserted into the MscI and XhoI restriction sites of the *pET-26b* vector to generate *rap-GS-pET26b*. Subsequently, the DDD2 coding sequence was amplified by PCR from *IFNα2b-DDD2-pdHL2*[33] and inserted into XhoI site of *rap-pET26b* to generate the expression vector *rap-GS-DDD2-pET26b*. Competent Rosetta-pLysS cells (EMD Millipore, Billerica, MA) were transformed with *rap-GS-DDD2-pET26b* and cultured in shaker flasks at 37°C in Difco 2xYT broth (Becton Dickinson, Franklin Lakes, NJ), supplemented with 100 μg/mL kanamycin sulfate and 34 μg/mL chloramphenicol. When the cell density reached OD_600_ = 1.0, cultures were switched to 30°C and protein expression was induced with 0.4 mM IPTG for 4 h. Cell pellets were frozen, thawed and homogenized in a lysis buffer comprising 2% Triton X-100, 5 mM MgSO_4_, 10 units/mL benzonase (Novagen EMD Millipore), 100 μM 4-(2-aminoethyl) benzenesulfonyl fluoride (Sigma-Aldrich, St. Louis, MO), and 20 mM Tris–HCl, pH 8.0. The insoluble material, containing inclusion bodies, was pelleted by centrifugation, re-homogenized in 1% Triton X-100 in PBS, and re-pelleted. Inclusion bodies were solubilized in 6 M guanidine-HCl, 100 mM Na-phosphate, pH 8.0, and applied to a His-Select affinity column (GE Healthcare, Piscataway, NJ). The denatured protein was eluted in 4 M guanidine-HCl, 100 mM NaH_2_PO_4_, pH 4.5. The eluate was neutralized with 3 M Tris–HCl, pH 8.6, added dithioerythreitol (DTE) to 60 mM, and held at room temperature overnight, to which was added rapidly 10-fold volume of 0.5 M arginine, 20 mM oxidized glutathione, 2 mM EDTA,100 mM Tris, pH 8.0, followed by dialysis against 5 L of a renaturation buffer (0.5 M arginine, 2 mM oxidized glutathione, 0.6 mM DTE, 2 mM EDTA, 20 mM Tris, pH 8.0) for 72 h at 4°C, and subsequently against PBS buffer.

Expression vectors for the C_H_3-AD2-IgG modules were engineered from cognate *IgG-pdHL2* expression vectors, as described previously [39]. The C_K_-AD2-IgG modules were generated by fusing AD2 and a hinge linker sequence to the C-terminal end of the *kappa* light chain [35]. These modules were produced in myeloma cell culture of SpESFX-10 cells [40] and isolated from culture broths using MabSelect affinity chromatography (GE Healthcare).

### DNL conjugation

The Rap-DDD2 module was reacted with a C_H_3-AD2-IgG or a C_K_-AD2-IgG of choice to generate a panel of DNL-Rap conjugates, as listed in Table 1, with the structures of the C_H_3- and C_K_-types shown schematically in Figure 1. Typically, an AD2-IgG was combined with approximately two mole-equivalents of Rap-DDD2, followed by the addition of reduced glutathione to a final concentration of 2 mM. After incubation at room temperature overnight, oxidized glutathione was added to a final concentration of 4 mM on the next day, the incubation continued for another 24 h, and the resulting DNL-Rap conjugate was purified by MabSelect affinity chromatography.

### Molecular characterization of DNL-Rap conjugates

SDS-PAGE was performed under reducing and non-reducing conditions using 4-20% Tris-Glycine gels (Lonza, Allendale, NJ). SE-HPLC was performed at the flow rate 1 ml/min and detection wavelength 280 nm using an Alliance HPLC System with a BioSuite 250, 4-μm UHR SEC column (Waters Corp. Milford, MA). Particle size analysis was performed by a contract laboratory (Microtrac, Largo, FL).

### Cell lines

All cell lines were purchased from the American Type Culture Collection (ATCC, Manassas, VA), which include seven of breast cancer (MCF-7, MDA-MB-468, MDA-MB-231, HCC1806, HCC1395, BT-20, SKBR-3), three of prostate cancer (MDA PCa 2b, PC-3, 22Rv1), one of pancreatic cancer (BxPC-3), one of lung cancer (Calu-3), one of cervical cancer (ME-180), and one of non-Hodgkin lymphoma (Daudi). Each cell line was maintained according to the recommendations of ATCC and routinely tested for mycoplasma using MycoAlert® Mycoplasma Detection Kit (Lonza).

### Internalization studies by fluorescence microscopy

MDA-MB-468 cells were placed at 3000 cells/well in 8-well, Lab-Tek II chamber slides (Nalge Nunc International) and incubated with 40 nM of (Rap)_2_-E1-(Rap)_2_ and 1:200 diluted human transferrin (hTf) conjugated to Alexa Fluor 568 (T-23365, Life Technologies Corporation) at 37°C for 2 h. After washing twice in PBS plus 2% BSA, cells were treated in 0.1 M glycine buffer (pH 2.5) for 2 min, and then fixed in 4% formalin for 15 min, followed by Alexa Fluor 488–conjugated goat anti-human IgG for 15 min. After washing twice with PBS, cells were stained with Hoechst 33258 for 5 min and examined under a fluorescence microscope.

### Flow cytometry

Cells were trypsinized briefly, washed, re-suspended in 1% BSA-PBS, incubated with hRS7 or hA20 IgG, and detected with FITC-GAH. All incubations were 45 min at 4°C with 1% BSA-PBS washes between incubations. Cell binding was measured by flow cytometry using a BD FACSCalibur (BD Biosciences, San Jose, CA).

### In vitro proliferation

Cells were seeded in 96-well plates at 1,000-2,000 cells/well and held at 37°C overnight prior to incubation with the indicated agents for 4 days. Viable cells were measured using MTS substrate Cell Titer96® AQueous One Solution (Promega, Madison, WI).

### Toxicity in human PBMC

Buffy coats from healthy donors were purchased from the Blood Center of New Jersey, with approval by the New England Institutional Review Board. PBMCs were isolated from buffy coats by standard density-gradient centrifugation over Ficoll-Paque, and treated with (Rap)_2_-E1-(Rap)_2_ at 37°C in 5% CO_2_ for 16 h. After incubation, the cells were stained with Alexa Fluor® 488-labeled annexin V, then with 7-aminoactinomycin-D, and analyzed by flow cytometry.

### Determination of maximal tolerated dose (MTD) in mice

Female athymic nude mice (8 weeks old) were divided into four groups of three each, with each group receiving (Rap)_2_-22*-(Rap)_2_ at one of four different doses (20, 40, 60 or 80 μg). Animals were dosed i.v. every four days for a total of four injections (q4dx4). Toxicity was assessed by daily observations and weights taken twice weekly. Mice were deemed to have lethal toxicity if they lost more than 15% of starting body weight or were otherwise deemed moribund. The MTD was the highest dose administered in which all the mice in the group survived the injections.

### In vivo therapeutic activity

All animal studies were approved by the University of Medicine and Dentistry of New Jersey’s Institutional Animal Care and Use Committee (IACUC) and performed in accordance with the Association for Assessment and Accreditation of Laboratory Animal Care, U.S. Department of Agriculture, and Department of Health and Human Services regulations. Female NCr athymic nude (*nu*/*nu*) mice, 5 weeks old, and SCID mice, 7 weeks old, were purchased from Taconic Farms (Hudson, NY). Two tumor models, solid and liquid cancers, were used to evaluate the efficacy and specificity of DNL-generated immunoRNases, (Rap)_2_-E1*-(Rap)_2_ and (Rap)_2_-22*-(Rap)_2_, as binding and nonbinding agents for MDA-MB-468 and vice versa for Daudi, respectively. For the MDA-MB-468 xenograft model, nude mice were each injected s.c. in the flank with 200 μL of MDA-MB-468 cells (1 × 10^7^) mixed 1:1 with matrigel (BD Bioscience). Once tumors reached approximately 0.2 cm^3^ in size, the animals were divided into three groups of 8 to 9 each. The treatment group received i.v. administration of the MTD of (Rap)_2_-E1*-(Rap)_2_ (20 μg i.v. q4dx4), followed by a second cycle 24 days after the last injection of the first cycle. The control group received the same dose/schedule of a CD22-targeting (Rap)_2_-22*-(Rap)_2_, which served as an alternative non-specific counterpart of (Rap)_2_-E1*-(Rap)_2_. The third group remained untreated and only received saline (100 μl). After treatment commenced, mice were weighed and tumors measured weekly. For both models, tumor volume (TV) was determined by measurements in two dimensions using calipers, with volumes defined as: *L* × *w*^2^/2, where *L* is the longest dimension of the tumor and *w* the shortest. When a tumor in individual mice ulcerated or exceeded 1.0 cm^3^ in measured volume, the animal was deemed to have succumbed to disease progression and was euthanized.

The model for disseminating human Burkitt lymphoma was established by injecting SCID mice i.v. with 1.5×10^7^ Daudi cells. On the day cells were administered, the mice were randomized into groups of 5 each. Therapy began 7 days later. The treatment groups received (Rap)_2_-22*-(Rap)_2_ at either the MTD (20 μg, q4dx4) or at half that dose (10 μg q4dx4). For the three controls, one group received the same amount of (Rap)_2_-E1*-(Rap)_2_, another group received the parental hLL2 IgG/epratuzumab (25 μg, q4dx4), and the third group received only saline (100 μL). Mice were deemed to have succumbed to disease progression when hind-limb paralysis developed, or if they lost more than 15% of their initial body weight, or if they became otherwise moribund.

Statistical analysis for the tumor growth data was based on AUC as well as survival time. The profile of tumor growth in each mouse was obtained through linear curve modeling. As a consequence of incompleteness of some of the growth curves (due to deaths), statistical comparisons of AUC was only performed up to the time at which the first animal within a group was euthanized due to disease progression. An *f*-test was employed to determine equality of variance between groups prior to statistical analysis of growth curves. A two-tailed *t*-test was used to assess statistical significance (*P* ≤ 0.05) between treatment groups except for the saline control in which a one-tailed *t*-test was utilized. Survival studies were analyzed from Kaplan-Meier plots (log-rank analysis), using the Prism GraphPad Software (v4.03) software package (Advanced Graphics Software, Rancho Santa Fe, CA).

## Abbreviations

Rap: Ranpirnase; (Rap): Rap-DDD2; (Rap)2: Dimerized Rap-DDD2; DNL: DOCK-AND-LOCK; TNBC: Triple-negative breast cancer; ER: Estrogen receptor; PR: Progesterone receptor; HER2: Human epidermal growth factor receptor type 2; PARP: Poly ADP ribose polymerase; mTOR: Mammalian target of rapamycin; SE-HPLC: Size-exclusion high-performance liquid chromatography.

## Competing interests

All authors are employees of Immunomedics, Inc., or IBC Pharmaceuticals, Inc., or both, and have stocks or stock options of Immunomedics, Inc. IBC Pharmaceuticals, Inc. is a fully own subsidiary of Immunomedics, Inc.

## Authors’ contributions

DL designed and performed in vitro studies, analyzed the data, and drafted the manuscript. TMC designed in vivo studies and performed the statistical analysis. YW performed dynamic light scattering analysis and cell internalization assay. EAR participated in the development of methodology. DMG and CHC conceived and directed the study, and wrote the paper. All authors read and approved the final manuscript.

## Supplementary Material

Additional file 1: Figure S1 Molecular characterization of selected DNL-Rap conjugates. A. Reducing (lanes 1–3) and non-reducing (lanes 4–6) SDS-PAGE analyses of Rap-DDD2 (lanes 1 and 4), C_H_3-AD2-IgG-hRS7 (lanes 2 and 5), and (Rap)_2_-E1-(Rap)_2_ (lanes 3 and 6). B. SE-HPLC profiles of (Rap)_2_-E1-(Rap)_2_, C_H_3-AD2-IgG-hRS7 (denoted as hRS7-IgG-AD2) and Rap-DDD2. C. Dynamic light scattering analysis of (Rap)_2_-E1-(Rap)_2_. D. SE-HPLC profiles of (Rap)_2_-E1*-(Rap)_2_ and (Rap)_2_-E1-(Rap)_2_. E. SDS-PAGE analyses of C_K_-AD2-IgG-hLL2, (Rap)_2_-22*-(Rap)_2_, Rap-DDD2, (Rap)_2_-22-(Rap)_2_, and C_H_3-AD2-IgG-hLL2. Click here for file

Additional file 2: Figure S2 Trop-2 expression in selected breast cancer cell lines as determined by binding to hRS7. Cells were incubated without or with 10 μg/mL of hRS7 or hA20 IgG on ice for 45 min, followed by FITC labeled goat anti-human IgG (GAH-FITC), and analyzed by flow cytometry. Data were processed by FlowJo software, with the MFI shown in Additional file 3: Table S1. Click here for file

Additional file 3: Table S1 Surface Trop-2 expression on selective breast cancer cell lines as determined by binding to hRS7. Click here for file

Additional file 4: Figure S3 *In vitro* cytotoxicity of (Rap)_2_-E1-(Rap)_2_ against a variety of solid cancer lines. Cells were harvested, plated into 96-well plates, and incubated with (Rap)_2_-E1-(Rap)_2_ at a final concentration ranging from 100 to 1 × 10^-6^ nM or 66.7 to 6.67×10^-7^ nM for a period of four doubling times, which were predetermined to be 7, 5, 5, 8, 5, and 6 days for MDA PCa 2b, PC-3, 22Rv1, Calu-3, BxPC-3, and ME-180, respectively. After each incubation period, MTS substrate (Cell Titer 96® AQueous One Solution; Promega) was added to all the wells and the color developed was measured at 1-h intervals for up to 4 h. Activity of test agents was calculated as a percent viability of treated cells relative to untreated cells using Microsoft Excel and Prism software (X = log [X]; non-linear regression sigmoidal dose response curves). As controls, cells were treated similarly with (Rap)_2_-22-(Rap)_2_, hRS7 IgG alone, or a combination of hRS7 IgG with either recombinant Rap (rRap, Ref. [36]) or Rap-DDD2. The EC_50_ values of (Rap)_2_-E1-(Rap)_2_ were determined to be 0.005, 0.04, 0.307, 0.032, 0.522, and >100 nM for MDA PCa 2b, PC-3, BxPC-3, ME-180, Calu-3, and 22Rv1, respectively. Click here for file

Additional file 5: Figure S4 Effect of (Rap)_2_-E1-(Rap)_2_ on hematological cells. Minimal toxicity of (Rap)_2_-E1-(Rap)_2_ to PBMCs from two healthy donors was indicated by comparable viable and apoptotic cells with those of untreated controls. Click here for file

Additional file 6: Figure S5 Tolerability of mice treated with two cycles of (Rap)_2_-E1*-(Rap)_2_ or (Rap)_2_-22*-(Rap)_2_ at MTD as assessed by the percent of starting body weight. Click here for file
