# Supplementary material for: Trop-2-targeting tetrakis-ranpirnase has potent antitumor activity against triple-negative breast cancer
Source: Mol Cancer. 2014 Mar 10;13:53. doi: 10.1186/1476-4598-13-53 (PMC4015355; doi:10.1186/1476-4598-13-53)
Supplement: Additional file 1: Figure S1 — Molecular characterization of selected DNL-Rap conjugates. A. Reducing (lanes 1–3) and non-reducing (lanes 4–6) SDS-PAGE analyses of Rap-DDD2 (lanes 1 and 4), CH3-AD2-IgG-hRS7 (lanes 2 and 5), and (Rap)2-E1-(Rap)2 (lanes 3 and 6). B. SE-HPLC profiles of (Rap)2-E1-(Rap)2, CH3-AD2-IgG-hRS7 (denoted as hRS7-IgG-AD2) and Rap-DDD2. C. Dynamic light scattering analysis of (Rap)2-E1-(Rap)2. D. SE-HPLC profiles of (Rap)2-E1*-(Rap)2 and (Rap)2-E1-(Rap)2. E. SDS-PAGE analyses of CK-AD2-IgG-hLL2, (Rap)2-22*-(Rap)2, Rap-DDD2, (Rap)2-22-(Rap)2, and CH3-AD2-IgG-hLL2. [file 1476-4598-13-53-S1.ppt]

## Slide 1
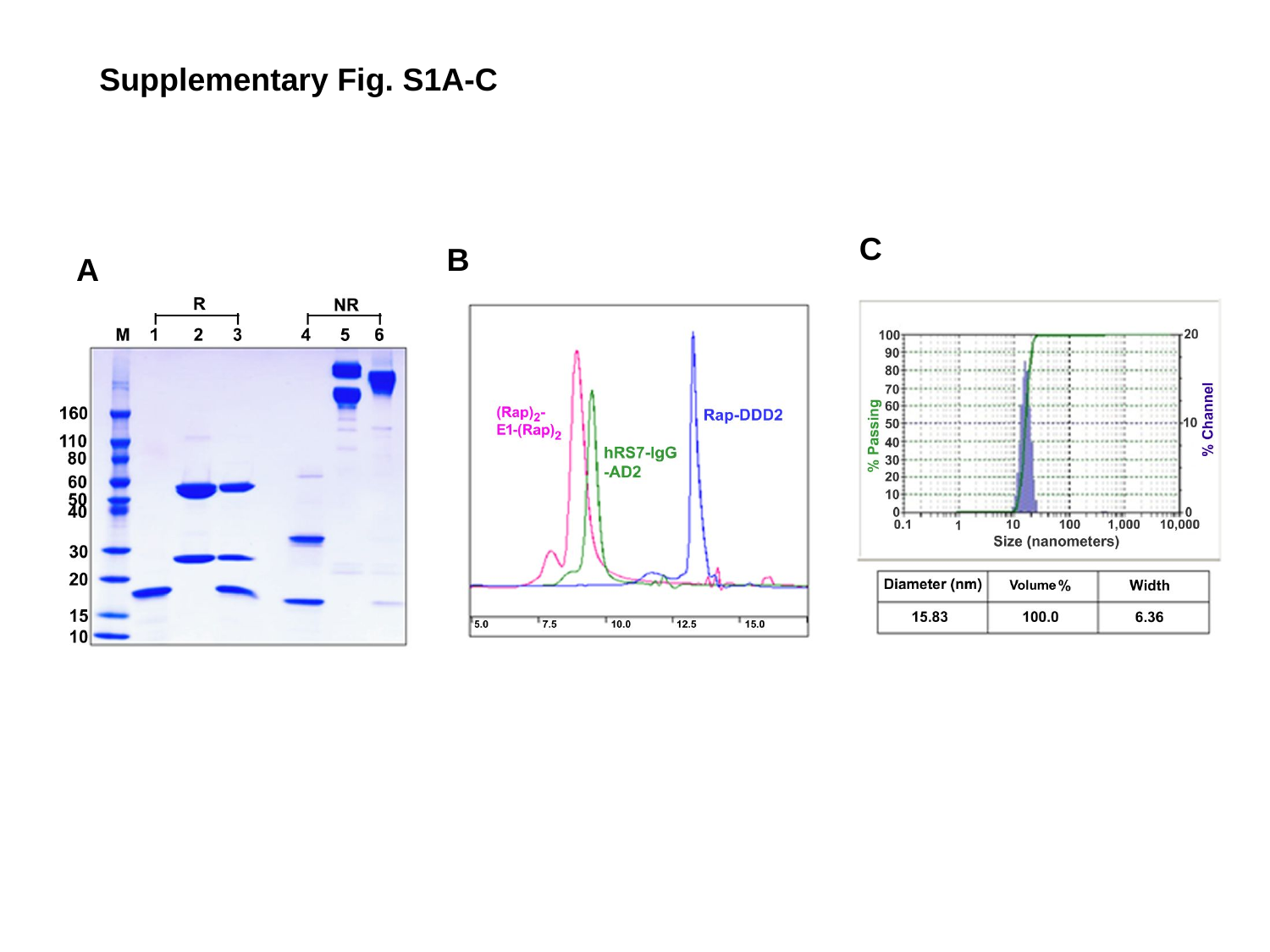

Supplementary Fig. S1A-C
C
B
A

## Slide 2
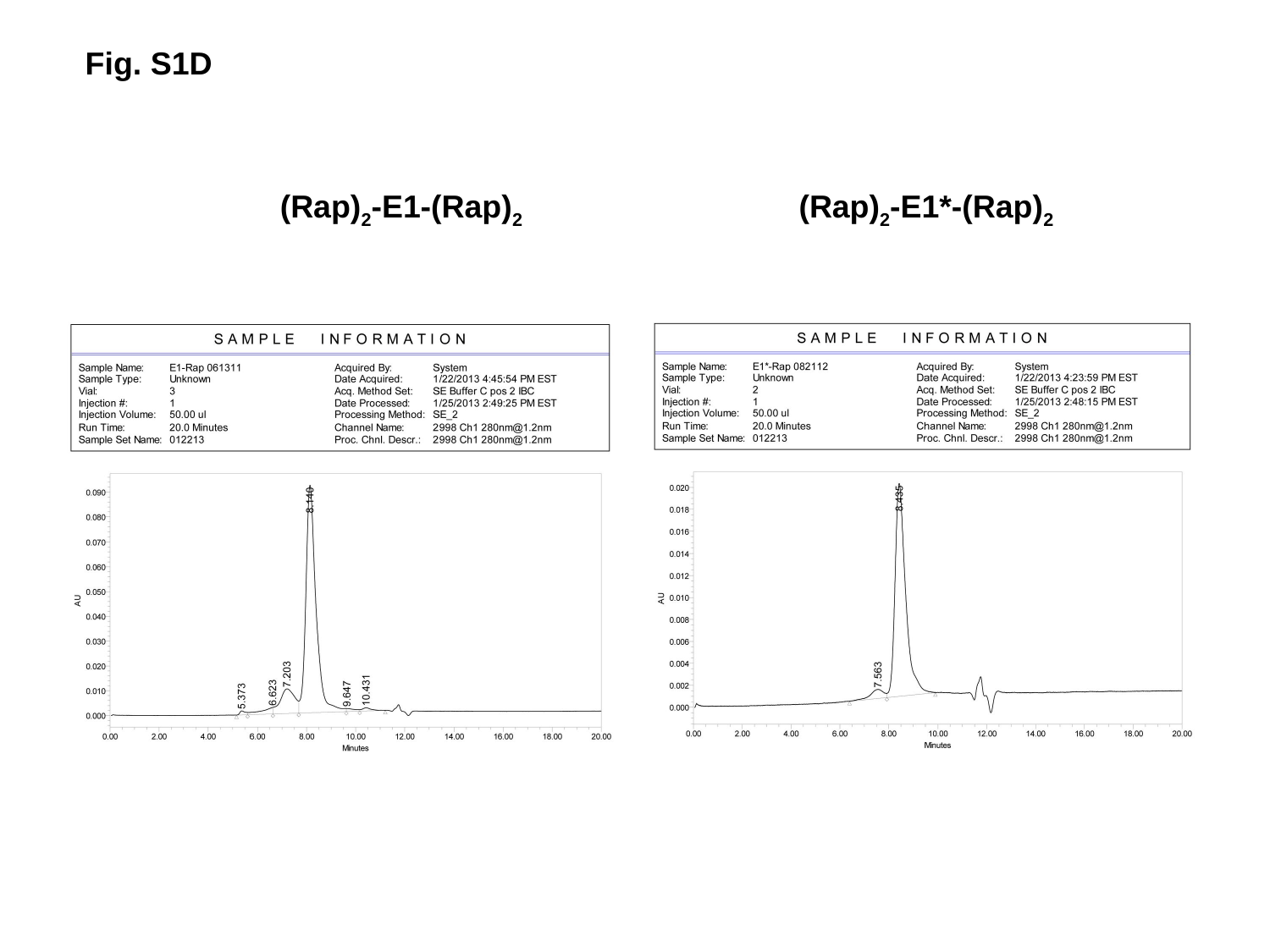

Fig. S1D
(Rap)2-E1-(Rap)2
(Rap)2-E1*-(Rap)2

## Slide 3
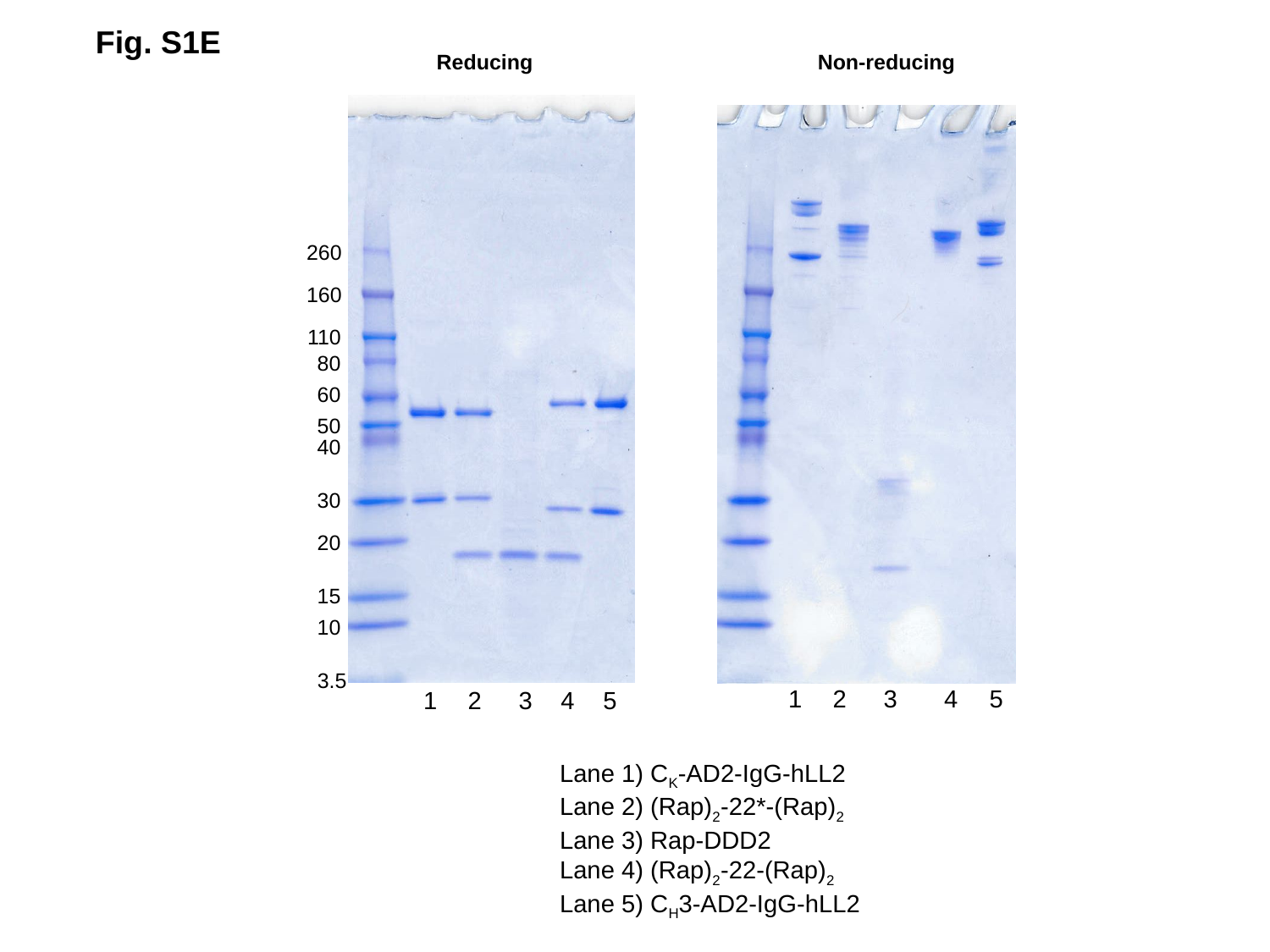

Fig. S1E
Reducing
Non-reducing
260
160
#
110
80
60
50
40
30
20
15
10
3.5
1
2
3
4
5
1
2
3
4
5
Lane 1) CK-AD2-IgG-hLL2
Lane 2) (Rap)2-22*-(Rap)2
Lane 3) Rap-DDD2
Lane 4) (Rap)2-22-(Rap)2
Lane 5) CH3-AD2-IgG-hLL2
