# Supplementary material for: Trop-2-targeting tetrakis-ranpirnase has potent antitumor activity against triple-negative breast cancer
Source: Mol Cancer. 2014 Mar 10;13:53. doi: 10.1186/1476-4598-13-53 (PMC4015355; doi:10.1186/1476-4598-13-53)
Supplement: Additional file 2: Figure S2 — Trop-2 expression in selected breast cancer cell lines as determined by binding to hRS7. Cells were incubated without or with 10 μg/mL of hRS7 or hA20 IgG on ice for 45 min, followed by FITC labeled goat anti-human IgG (GAH-FITC), and analyzed by flow cytometry. Data were processed by FlowJo software, with the MFI shown in Additional file 3: Table S1. [file 1476-4598-13-53-S2.ppt]

## Slide 1
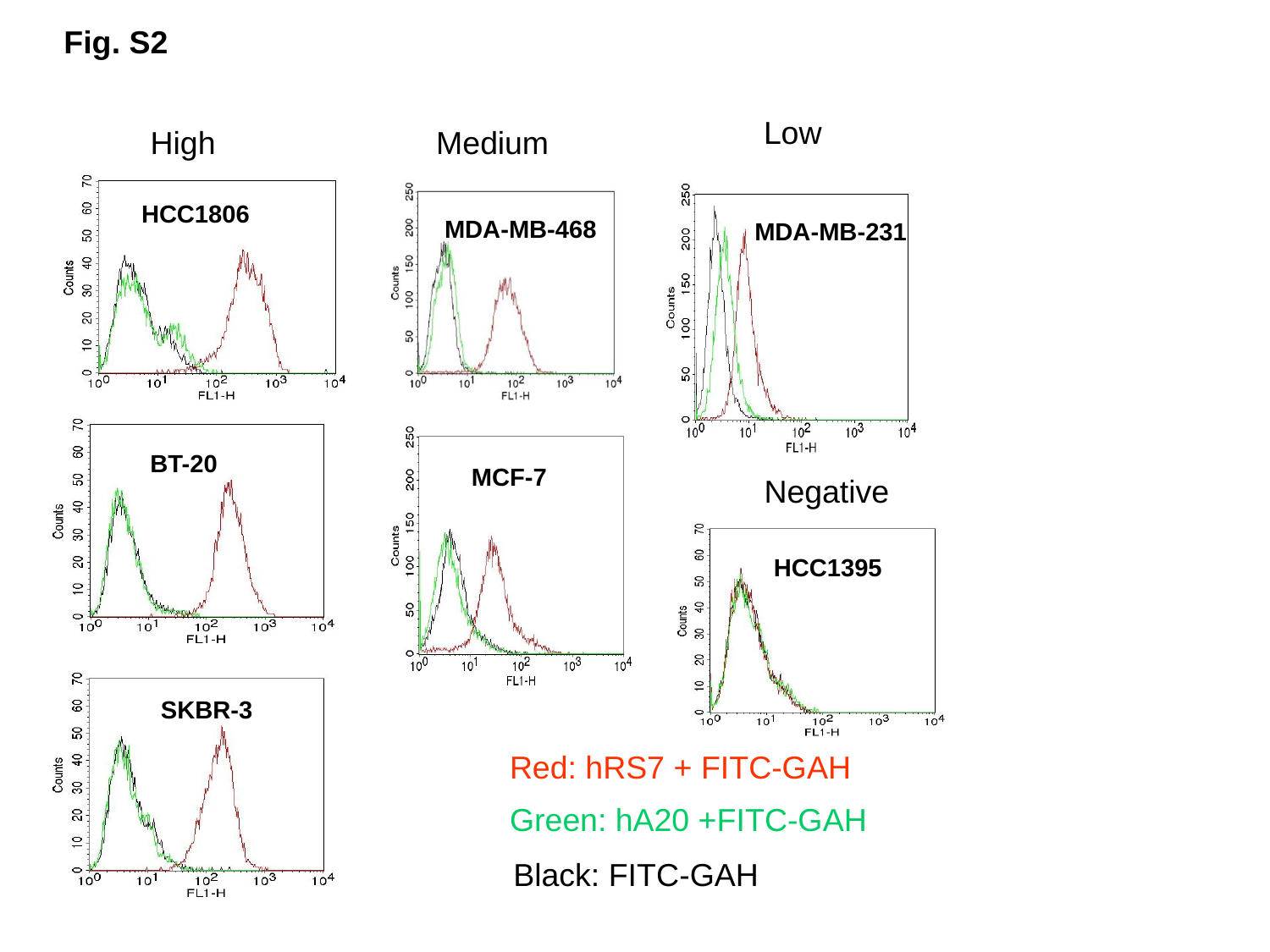

Fig. S2
Low
High
Medium
HCC1806
MDA-MB-468
MDA-MB-231
BT-20
MCF-7
Negative
HCC1395
SKBR-3
Red: hRS7 + FITC-GAH
Green: hA20 +FITC-GAH
Black: FITC-GAH
