# Supplementary material for: Trop-2-targeting tetrakis-ranpirnase has potent antitumor activity against triple-negative breast cancer
Source: Mol Cancer. 2014 Mar 10;13:53. doi: 10.1186/1476-4598-13-53 (PMC4015355; doi:10.1186/1476-4598-13-53)
Supplement: Additional file 3: Table S1 — Surface Trop-2 expression on selective breast cancer cell lines as determined by binding to hRS7. [file 1476-4598-13-53-S3.doc]

**Additional file 3:** Table S1. Surface Trop-2 expression on selective breast cancer cell lines as determined by binding to hRS7.

|  | **Median fluorescence intensity (MFI)** | | | | | | |
| --- | --- | --- | --- | --- | --- | --- | --- |
|  | HCC1806 | BT-20 | SKBR-3 | MDA-MB-468 | MCF-7 | MDA-MB-231 | HCC1395 |
| GAH-FITC | 11.26 | 5.30 | 6.31 | 3.39 | 5.79 | 2.88 | 7.23 |
| +hA20 | 9.48 | 5.23 | 6.26 | 4.13 | 5.31 | 4.05 | 7.04 |
| +hRS7 | 361.43 | 288.78 | 176.53 | 75.80 | 43.01 | 10.37 | 6.30 |
