# Supplementary material for: Trop-2-targeting tetrakis-ranpirnase has potent antitumor activity against triple-negative breast cancer
Source: Mol Cancer. 2014 Mar 10;13:53. doi: 10.1186/1476-4598-13-53 (PMC4015355; doi:10.1186/1476-4598-13-53)
Supplement: Additional file 4: Figure S3 — In vitro cytotoxicity of (Rap)2-E1-(Rap)2 against a variety of solid cancer lines. Cells were harvested, plated into 96-well plates, and incubated with (Rap)2-E1-(Rap)2 at a final concentration ranging from 100 to 1 × 10^-6^ nM or 66.7 to 6.67×10^-7^ nM for a period of four doubling times, which were predetermined to be 7, 5, 5, 8, 5, and 6 days for MDA PCa 2b, PC-3, 22Rv1, Calu-3, BxPC-3, and ME-180, respectively. After each incubation period, MTS substrate (Cell Titer 96® AQueous One Solution; Promega) was added to all the wells and the color developed was measured at 1-h intervals for up to 4 h. Activity of test agents was calculated as a percent viability of treated cells relative to untreated cells using Microsoft Excel and Prism software (X = log [X]; non-linear regression sigmoidal dose response curves). As controls, cells were treated similarly with (Rap)2-22-(Rap)2, hRS7 IgG alone, or a combination of hRS7 IgG with either recombinant Rap (rRap, Ref. [36]) or Rap-DDD2. The EC50 values of (Rap)2-E1-(Rap)2 were determined to be 0.005, 0.04, 0.307, 0.032, 0.522, and >100 nM for MDA PCa 2b, PC-3, BxPC-3, ME-180, Calu-3, and 22Rv1, respectively. [file 1476-4598-13-53-S4.ppt]

## Slide 1
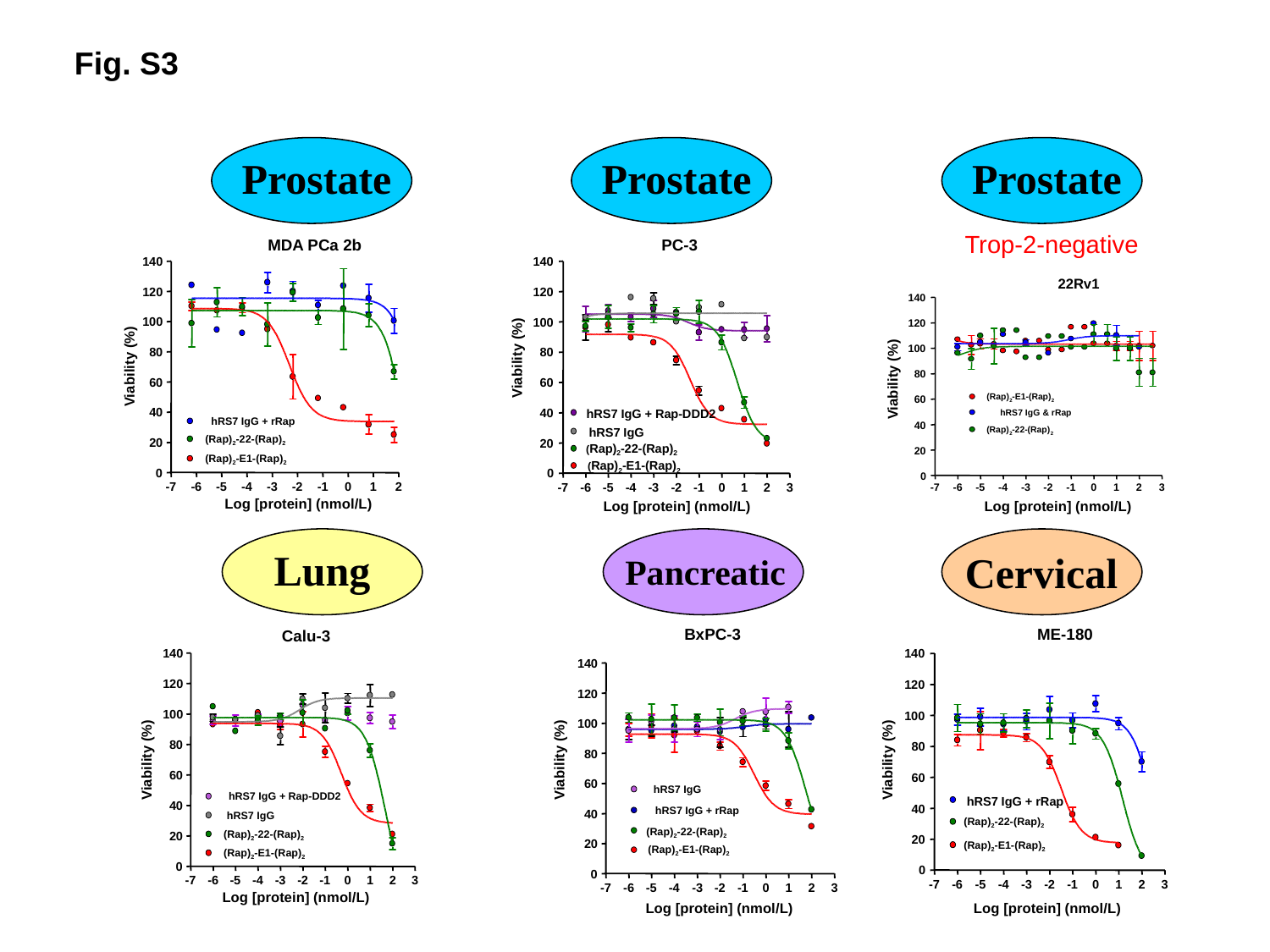

Fig. S3
Prostate
Prostate
Prostate
 MDA PCa 2b
140
120
100
80
Viability (%)
60
40
hRS7 IgG + rRap
(Rap)2-22-(Rap)2
20
(Rap)2-E1-(Rap)2
0
-7
-6
-5
-4
-3
-2
-1
0
1
2
Log [protein] (nmol/L)
PC-3
140
120
100
80
60
40
hRS7 IgG + Rap-DDD2
hRS7 IgG
20
0
-7
-6
-5
-4
-3
-2
-1
0
1
2
3
Trop-2-negative
 22Rv1
-7
140
120
100
80
(Rap)2-E1-(Rap)2
60
hRS7 IgG & rRap
40
(Rap)2-22-(Rap)2
20
0
-6
-5
-4
-3
-2
-1
0
1
2
3
Viability (%)
Viability (%)
(Rap)2-22-(Rap)2
(Rap)2-E1-(Rap)2
Log [protein] (nmol/L)
Log [protein] (nmol/L)
Lung
Pancreatic
Cervical
Calu-3
140
120
100
80
60
hRS7 IgG + Rap-DDD2
40
hRS7 IgG
(Rap)2-22-(Rap)2
20
(Rap)2-E1-(Rap)2
0
-7
-6
-5
-4
-3
-2
-1
0
1
2
3
 ME-180
-7
-6
-5
-4
-3
-2
-1
0
1
2
3
140
120
100
80
60
hRS7 IgG + rRap
40
20
0
 BxPC-3
140
120
100
80
60
hRS7 IgG
hRS7 IgG + rRap
40
20
0
-7
-6
-5
-4
-3
-2
-1
0
1
2
3
Viability (%)
Viability (%)
Viability (%)
(Rap)2-22-(Rap)2
(Rap)2-22-(Rap)2
(Rap)2-E1-(Rap)2
(Rap)2-E1-(Rap)2
Log [protein] (nmol/L)
Log [protein] (nmol/L)
Log [protein] (nmol/L)
