# Supplementary figures and images for: Trop-2-targeting tetrakis-ranpirnase has potent antitumor activity against triple-negative breast cancer
Source: Mol Cancer. 2014 Mar 10;13:53. doi: 10.1186/1476-4598-13-53 (PMC4015355; doi:10.1186/1476-4598-13-53)

## Slide 1
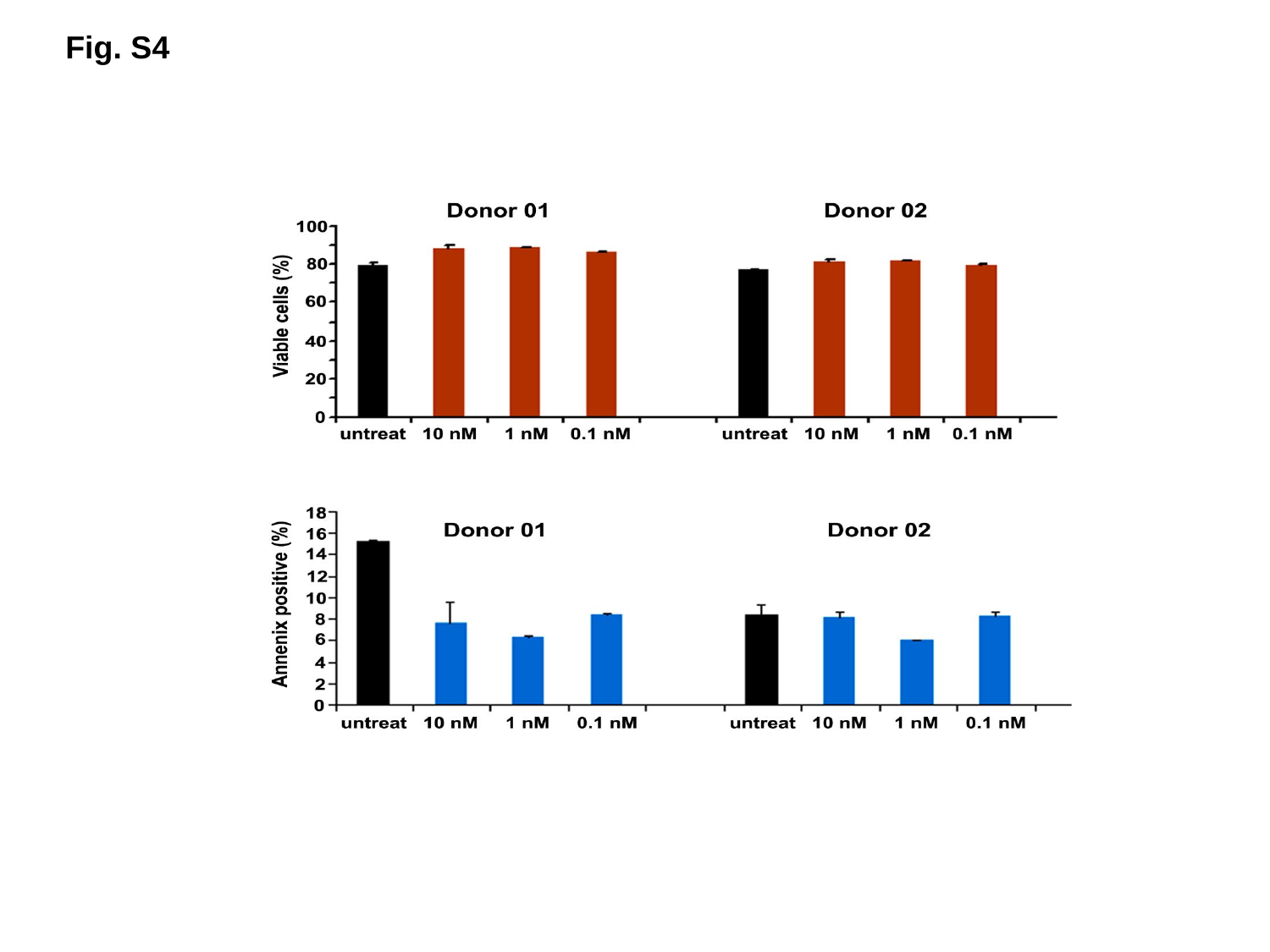

Fig. S4

Supplement: Additional file 5: Figure S4 — Effect of (Rap)2-E1-(Rap)2 on hematological cells. Minimal toxicity of (Rap)2-E1-(Rap)2 to PBMCs from two healthy donors was indicated by comparable viable and apoptotic cells with those of untreated controls. [file 1476-4598-13-53-S5.ppt]
