# Supplementary material for: Trop-2-targeting tetrakis-ranpirnase has potent antitumor activity against triple-negative breast cancer
Source: Mol Cancer. 2014 Mar 10;13:53. doi: 10.1186/1476-4598-13-53 (PMC4015355; doi:10.1186/1476-4598-13-53)
Supplement: Additional file 6: Figure S5 — Tolerability of mice treated with two cycles of (Rap)2-E1*-(Rap)2 or (Rap)2-22*-(Rap)2 at MTD as assessed by the percent of starting body weight. [file 1476-4598-13-53-S6.ppt]

## Slide 1
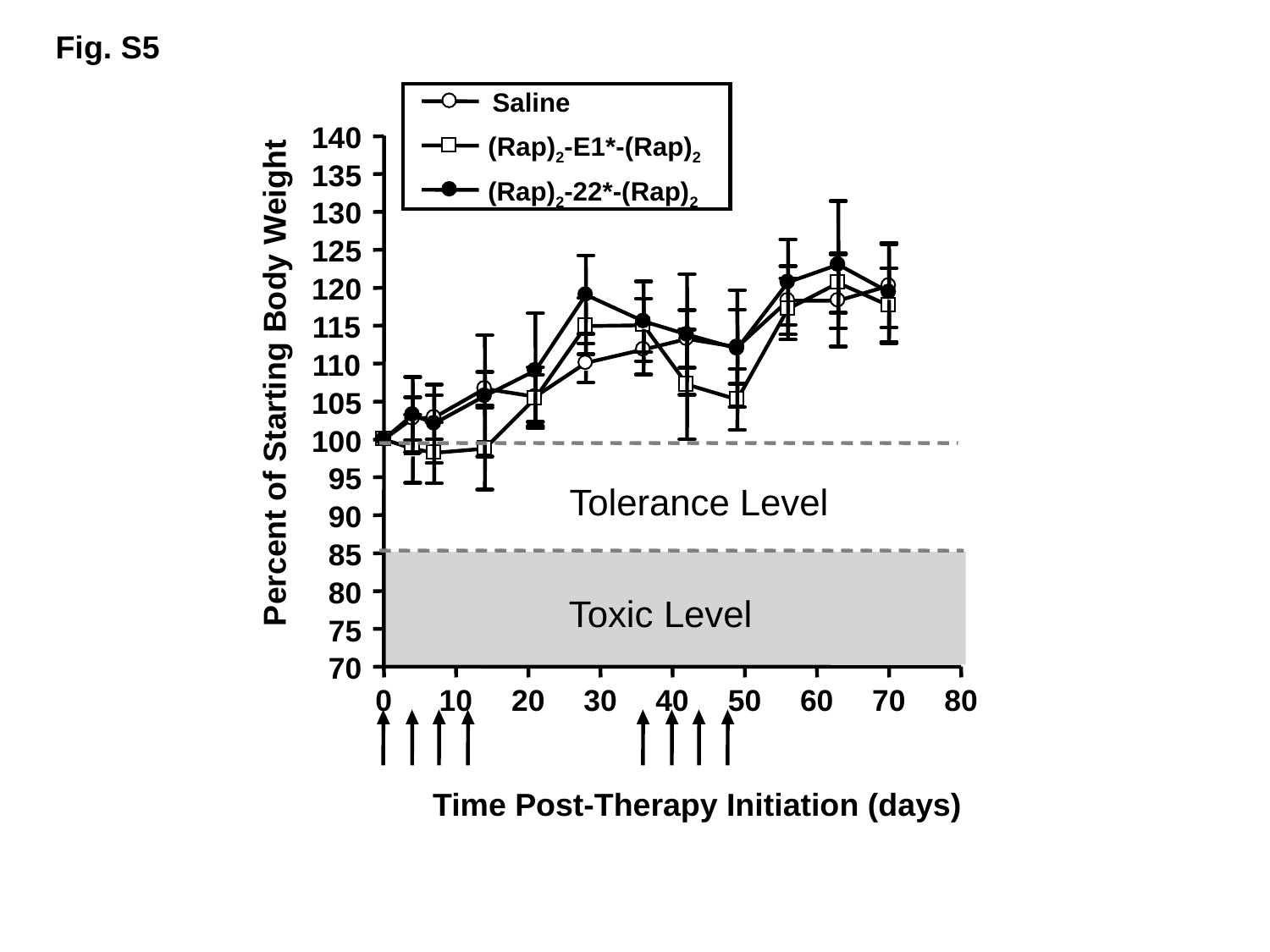

Fig. S5
Saline
140
(Rap)2-E1*-(Rap)2
135
(Rap)2-22*-(Rap)2
130
125
120
115
110
Percent of Starting Body Weight
105
100
95
Tolerance Level
90
85
80
Toxic Level
75
70
0
10
20
30
40
50
60
70
80
Time Post-Therapy Initiation (days)
